# Comparison of principal component analysis algorithms for imputation in agrometeorological data in high dimension and reduced sample size

**DOI:** 10.1371/journal.pone.0315574

**Published:** 2024-12-31

**Authors:** Valter Cesar de Souza, Sergio Augusto Rodrigues, Luís Roberto Almeida Gabriel Filho

**Affiliations:** 1 São Paulo State University (Unesp), School of Agriculture, Botucatu, São Paulo, Brasil; 2 São Paulo State University (Unesp), School of Sciences and Engineering, Tupã, São Paulo, Brasil; University 20 Aout 1955 skikda, Algeria, ALGERIA

## Abstract

Meteorological data acquired with precision, quality, and reliability are crucial in various agronomy fields, especially in studies related to reference evapotranspiration (ETo). ETo plays a fundamental role in the hydrological cycle, irrigation system planning and management, water demand modeling, water stress monitoring, water balance estimation, as well as in hydrological and environmental studies. However, temporal records often encounter issues such as missing measurements. The aim of this study was to evaluate the performance of alternative multivariate procedures for principal component analysis (PCA), using the Nonlinear Iterative Partial Least Squares (NIPALS) and Expectation-Maximization (EM) algorithms, for imputing missing data in time series of meteorological variables. This was carried out on high-dimensional and reduced-sample databases, covering different percentages of missing data. The databases, collected between 2011 and 2021, originated from 45 automatic weather stations in the São Paulo region, Brazil. They were used to create a daily time series of ETo. Five scenarios of missing data (10%, 20%, 30%, 40%, 50%) were simulated, in which datasets were randomly withdrawn from the ETo base. Subsequently, imputation was performed using the NIPALS-PCA, EM-PCA, and simple mean imputation (IM) procedures. This cycle was repeated 100 times, and average performance indicators were calculated. Statistical performance evaluation utilized the following indicators: correlation coefficient (r), Mean Absolute Error (MAE), Mean Absolute Percentage Error (MAPE), Mean Square Error (MSE), Normalized Root Mean Square Error (nRMSE), Willmott Index (d), and performance index (c). In the scenario with 10% missing data, NIPALS-PCA achieved the lowest MAPE (15.4%), followed by EM-PCA (17.0%), while IM recorded a MAPE of 24.7%. In the scenario with 50% missing data, there was a performance reversal, with EM-PCA showing the lowest MAPE (19.1%), followed by NIPALS-PCA (19.9%). The NIPALS-PCA and EM-PCA approaches demonstrated good results in imputation (10% ≤ nRMSE < 20%), with NIPALS-PCA excelling in the 10%, 20%, and 30% scenarios, and EM-PCA in the 40% and 50% scenarios. Based on statistical evaluation, the NIPALS-PCA, EM-PCA, and IM imputation models proved suitable for estimating missing ETo data, with PCA imputation models in the NIPALS and EM algorithms showing the most promise. Future research should explore the effectiveness of various imputation methods in diverse climatic and geographical contexts, as well as develop new techniques considering the temporal and spatial structure of meteorological data, to advance understanding and climate prediction.

## Introduction

Measuring evapotranspiration represents a significant challenge in agricultural meteorology [1, 2], mainly due to the high costs associated with direct measurement techniques in terms of implementation, operation and maintenance of measuring equipment [3, 4]. As an alternative, indirect methods are used [5–10] by means of mathematical equations capable of adjusting to local climatic conditions, requiring historical series of meteorological data. However, data recorded over time is generally subject to flaws or errors [11], such as missing measurements, commonly referred to as missings [12].

Addressing missing data is crucial for accurate analysis and decision-making in meteorological studies. Among the various methods available for imputing missing data [13–22], Principal Component Analysis (PCA) has emerged as a versatile and effective tool for exploratory data analysis and characterization of spatial variability [23–25]. Traditional PCA, known for exploratory analysis and dimensionality reduction, can also serve as a viable imputation procedure for missing data [26]. Iterative methods such as the Nonlinear Iterative Partial Least Squares (NIPALS) algorithm [27–33] and the Expectation-Maximization (EM) algorithm [34–38] are commonly employed in conjunction with PCA for imputation.

The application of the NIPALS-PCA and EM-PCA algorithms to the imputation of missing data has shown promising results in various domains. For example, Martí and Zarzo [24] demonstrated the superiority of NIPALS-PCA in imputing reference evapotranspiration data recorded along the Mediterranean coast of Spain compared to methods based on nearest neighbors. Similarly, Josse and Husson [39] introduced the EM-PCA method in the missMDA package of the R-Gui computing environment [40], making it easier to reconstruct the data matrix for high-dimensional data sets [23, 41–44].

In this context, even considering the advances, there is still a need for further exploration and evaluation of alternative imputation techniques that employ PCA, particularly with regard to their performance in different domains and datasets [44]. This study aims to fill part of this gap by investigating the performance of alternative multivariate principal component analysis procedures (NIPALS-PCA and EM-PCA) in the imputation of missing data in time series of meteorological variables. Specifically, considering databases in high-dimensional scenarios and reduced sample size, evaluating their performance under different percentages of missing data.

## Material and methods

The research was structured in three phases, in which we sought to carry out an extensive literature review on the subject of Missing Value Imputation (MVI), detail an algorithm for how to carry out the simulation steps in MVI and compare alternative procedures for applying principal component analysis techniques in situations of high dimension, reduced sample size and lack of data, by evaluating performance against observed reference evapotranspiration data considering different contexts.

The phases were structured as follows: Phase 1: Understand the panorama of worldwide research on the subject of Missing Value Imputation through a bibliometric analysis; Phase 2: Detail a plan for simulation studies in MVI, focusing on the database and type of data, mechanism and missing rate, imputation technique and performance evaluation method, preparing concepts for the application, used in phase three; Phase 3: Compares the performance of alternative multivariate procedures of principal component analysis in the imputation of missing data in time series of meteorological variables, considering databases in the high-dimensional and reduced-sample scenario, with different rates of missing data.

**Fig 1:**
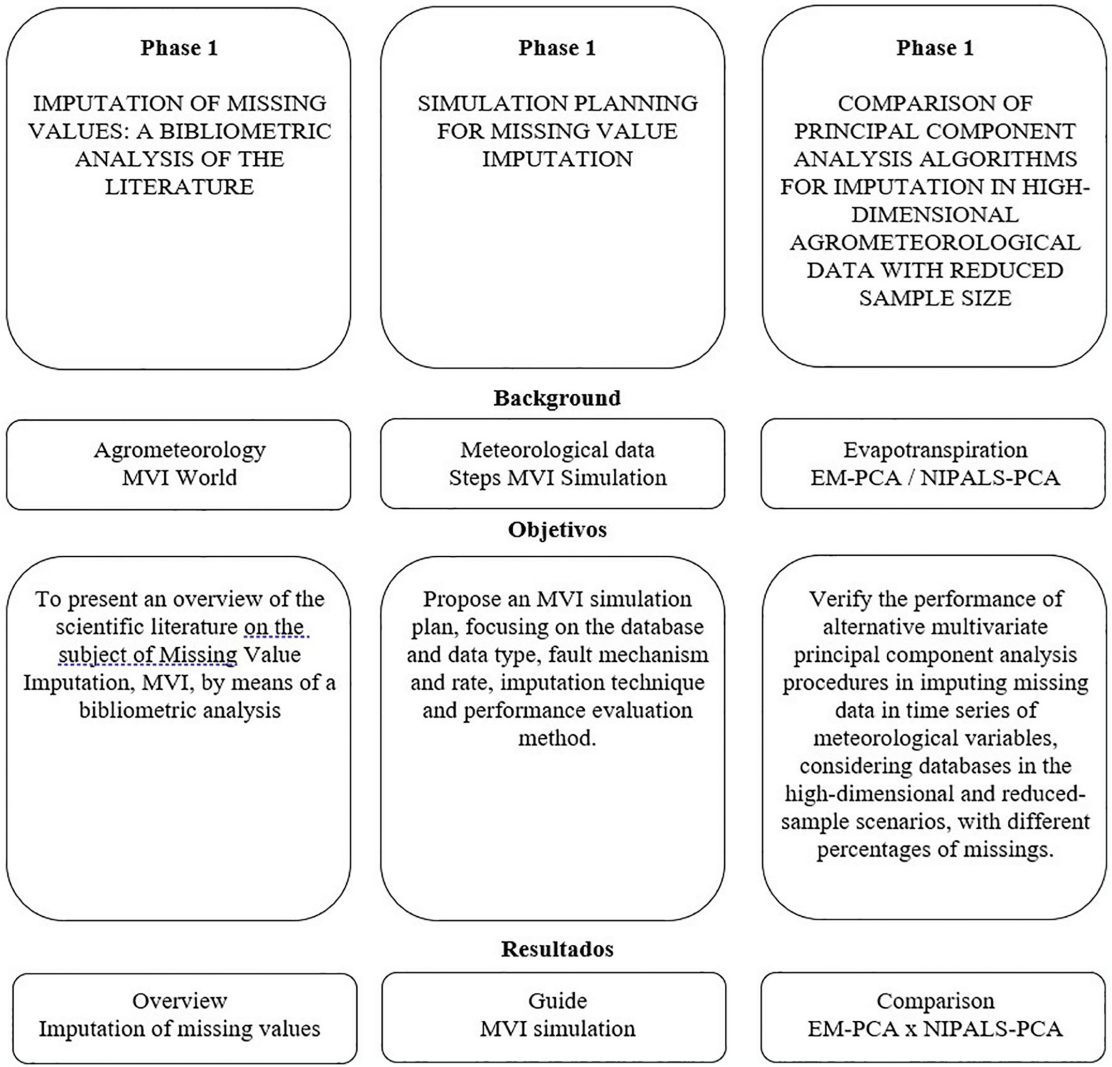
Research methodological framework. **Source**: Own authorship.

Fig 1 illustrates the methodological development used by means of a structured framework. It provides an overview of the organization of the phases, emphasizing the deliverables of each phase.

This paper focuses on phase three: comparison of principal component analysis algorithms for imputation in high-dimensional agrometeorological data with a reduced sample size.

### Simulation planning

**Fig 2:**
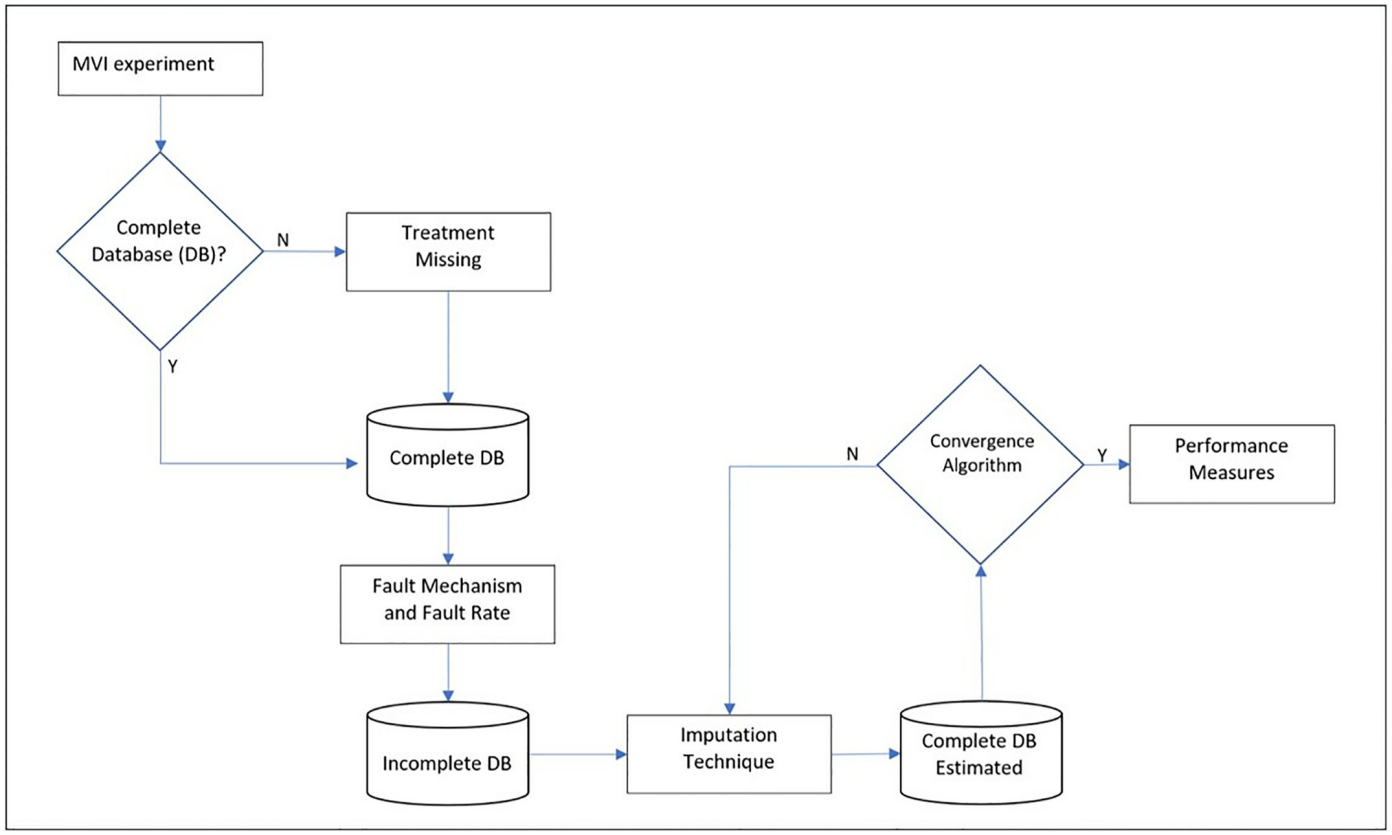
Steps in an MVI experiment. **Source**: Own authorship.

There are some important definitions and steps when planning an MVI simulation study, which are: database and data type, mechanism and fault rate, imputation technique and performance evaluation method. Simulation studies, as is the case in this paper, usually aim to verify the performance of imputation methods, as shown in Fig 2, considering the interactions between application type, data type, mechanism, and fault rate.

### Databases

Hourly databases were used, provided by *National Meteorological Institute* (INMET) [45] were used for each meteorological variable, from January 1, 2012, to December 31, 2021, evaluated at 45 automatic weather stations in the region of the State of São Paulo, Brazil.

For each station, the hourly databases covering the period in question were downloaded from the website of the *National Meteorological Institute* in.*csv* format files, totaling four hundred and fifty files (10 years x 45 stations).

### Extracting weather data from INMET

To download weather data from INMET’s historical series, several steps are required:

1. Log on to the INMET website: https://bdmep.inmet.gov.br/;
2. Choose the annual data package option for all automatic stations separated by year, and you will be taken to the page for annual historical data;
3. Choose the years of interest, among which data is available from the year 2000 onwards. For each year selected, a file in “.csv”, comma separated values, format will be available for each station;
4. Choose the stations of interest for the particular survey. To choose the stations of interest, view the geographical distribution of the stations on the map of stations on the link: https://mapas.inmet.gov.br/;
5. Rename all the files (.csv), this can be done manually or automatically. The automatic method is preferable due to the number of files to be handled by a data processing routine, for example, for a choice of 45 stations for a period of 10 years, there are 450 files. In order to merge and automatically process the data contained in these files, it is necessary to standardize the names. Going from a full name, for example, INMET_SE_SP_A725_AVARE_01-01-2011_A_31-12-2011 to a shortened name A725_2011;
6. To facilitate the routine reading of these files, create a folder for each station with the spreadsheet files for the years of interest;
7. Create a routine using a script in the ***R*** environment that automatically reads the data files obtained, considering the following steps:
  - Read the files with the data from each station for all the years of the research;
  - Exclude the first 9 lines, as they contain information on the weather stations from the research data source (INMET);
  - Use common column names for all the databases read into the ***R*** environment;
  - Replace all "-9999" values with "NA";
  - Convert the date-time (Greenwich time zone) to local time, with a specific adjustment for São Paulo, subtracting three hours.
  - Create a database aggregating all the files;
  - Recalculate the variables of interest on a daily basis.

### Automatic weather stations

Table 1 provides information on the automatic weather stations used in this research.

**Table 1:** Information on automatic weather stations.

ID	Location	State	Latitude [°]	Longitude [°]	Altitude [m]
1	ARIRANHA	SP	21°7’59’’S	48°50’26’’W	525,4
2	AVARE	SP	23°6’6’’S	48°56’28’’W	776,4
3	BARRA BONITA	SP	22°28’16’’S	48°33’27’’W	533,7
4	BARRA DO TURVO	SP	24°57’46’’S	48°24’59’’W	659,9
5	BARRETOS	SP	20°33’33’’S	48°32’42’’W	534,4
6	BARUERI	SP	23°31’26’’S	46°52’10’’W	776,5
7	BAURU	SP	22°21’29’’S	49°1’44’’W	636,2
8	JORDAN FIELDS	SP	22°45’1’’S	45°36’14’’W	1.663,0
9	WHITE HOUSE	SP	21°46’50’’S	47°4’31’’W	734,2
10	FRANCE	SP	20°35’4’’S	47°22’57’’W	1.002,8
11	IBITINGA	SP	21°51’20’’S	48°47’59’’W	496,8
12	IGUAPE	SP	24°40’18’’S	47°32’45’’W	2,7
13	ITAPEVA	SP	23°58’55’’S	48°53’9’’W	743,3
14	ITAPIRA	SP	22°24’54’’S	46°48’19’’W	634,9
15	ITUVERAVA	SP	20°21’35’’S	47°46’31’’W	610,6
16	JALES	SP	20°9’54’’S	50°35’42’’W	460,4
17	LINS	SP	21°39’58’’S	49°44’5’’W	460,7
18	PIRACICABA	SP	22°42’11’’S	47°37’24’’W	566,5
19	PRADOPOLIS	SP	21°20’18’’S	48°6’50’’W	540,4
20	PRESIDENT PRUDENTE	SP	22°7’12’’S	51°24’31’’W	431,9
21	RANCHARIA	SP	22°22’22’’S	50°58’29’’W	398,8
22	SAO CARLOS	SP	21°58’49’’S	47°53’2’’W	859,3
23	SAO LUIS PARAITINGA	SP	23°13’42’’S	45°25’1’’W	862,3
24	SAO MIGUEL ARCANJO	SP	23°51’7’’S	48°9’53’’W	675,7
25	SAO PAULO—MIRANTE	SP	23°29’47’’S	46°37’12’’W	785,6
26	SOROCABA	SP	23°25’34’’S	47°35’8’’W	609,3
27	TAUBATE	SP	23°2’30’’S	45°31’15’’W	582,3
28	VALPARAISO	SP	21°19’9’’S	50°55’49’’W	381,9
29	VOTUPORANGA	SP	20°24’12’’S	49°57’58’’W	510,4
30	PARATY	RJ	23°13’25’’S	44°43’37’’W	3,0
31	RESENDE	RJ	22°27’5’’S	44°26’42’’W	438,8
31	RESENDE	RJ	22°27’5’’S	44°26’42’’W	438,8
32	JAPIRA	PR	23°46’24’’S	50°10’50’’W	692,9
33	MARINGA	PR	23°24’19’’S	51°55’58’’W	548,5
34	NEW FATIMA	PR	23°24’55’’S	50°34’40’’W	664,3
35	PARANAPOEMA	PR	22°39’30’’S	52°8’4’’W	308,7
36	VENTANIA	PR	24°16’49’’S	50°12’37’’W	1.093,4
37	AGUA CLARA	MS	20°26’40’’S	52°52’33’’W	323,6
38	PARANAIBA	MS	19°41’44’’S	51°10’54’’W	408,1
39	CALDAS	MG	21°55’5’’S	46°22’59’’W	1.077,3
40	CAMPINA VERDE	MG	19°32’21’’S	49°31’5’’W	559,1
41	CONCEICAO DAS ALAGOAS	MG	19°59’9’’S	48°9’6’’W	572,5
42	MONTE VERDE	MG	22°51’42’’S	46°2’36’’W	1.544,9
43	PASS FOUR	MG	22°23’45’’S	44°57’43’’W	1.017,1
44	STEPS	MG	20°44’43’’S	46°38’2’’W	781,7
45	SACRAMENTO	MG	19°52’31’’S	47°26’3’’W	913,1

**Source**: Own authorship based on INMET data.

### Variables of interest

A routine was created in R [40] was created to aggregate and generate daily data for the variables of interest: global solar irradiation (***R***_***s***_, MJ m^-2^ hour^-1^), maximum and minimum air temperatures (***T***_***max***_ and ***T***_***min***_, °C), maximum and minimum relative humidity (***RH***_***max***_ and ***RH***_***min***_, %) and wind speed (***u***_***2***_, m s^-1^) measured at a height of 2 meters from the surface.

### Reference evapotranspiration

Next, with the daily values of the variables (***R***_***s***_, ***T***_***max***_, ***T***_***min***_, ***UR***_***max***_, ***UR***_***min***_, ***u***_***2***_) and using the Penman-Monteith model [46], recommended by the Food and Agriculture Organization, in bulletin FAO-56, a daily database of reference evapotranspiration (ET)_o_ was obtained for this region. Naturally, this initial database, a matrix of 45 stations (in rows) by 3653 days (in columns), totaling 164,385 elements, had missing data, about 9.45%, which corresponds to 15,531 missing data in total, which were fully filled in by the *average value of the column* corresponding to the position of *the* missing data, resulting in a complete database. This complete database was used to verify, using a *script* implemented in the ***R*** environment, the performance of the NIPALS-PCA algorithms [33, 47] EM-PCA [39] and imputation by the mean of the columns (IM) for filling in missing data, carried out by means of a simulation to evaluate the methods for imputing *missings* in the complete daily ET_o_ data matrix.

### Equação de penman-monteith

The Penman-Monteith model [46] for calculating reference evapotranspiration, ETo, given by the equation:

$$ET_{o}=\frac{0,408\;\Delta \;\left(R_{n}-G\right)+\gamma \;\frac{900}{T_{med}+273}\;u_{2}\left(e_{s}-e_{a}\right)}{\Delta +\gamma \left(1+0,34\;u_{2}\right)}$$ (1)

Where: *ET*_*o*_—reference evapotranspiration (mm dia^-1^), *Rn—*net radiation at the crop surface (MJ m^-2^ dia^-1^), *G—*soil heat flux density (MJ m^-2^ dia^-1^), *T*_*med*_—mean daily air temperature at 2 m height (°C), *u*_*2*_—wind speed at 2 m height (m s^-1^), *e*_*s*_—saturation vapour pressure (kPa), *e*_*a*_—actual vapour pressure (kPa), *e*_*s*_*—e*_*a*_—saturation vapour pressure déficit (kPa), Δ—slope vapour pressure curve (kPa ºC^-1^), γ—psychrometric constant (kPa ºC^-1^).

### Imputation methods

The treatment of missing data can begin with the decision to eliminate or estimate the missing values [48]. To eliminate missing values, techniques such as complete deletion (listwise deletion) and pairwise deletion are used. In listwise deletion, all cases with at least one missing value are eliminated, which can result in the loss of a lot of data.

In pairwise deletion, only observations with missing values for the variable of interest are excluded, which allows different subsets of data to be used in different analyses, depending on the availability of data for each variable. Although paired exclusion can be more efficient than complete exclusion of cases, it can result in different sample sizes for each analysis and affect the validity of comparisons between variables. These approaches are simple and easy to implement but can result in significant loss of information [49].

Imputation refers to replacing missing data with estimated values. There are several ways in which missing values can be imputed, depending on the nature of the problem and the data. Depending on the nature of the problem, imputation techniques can be broadly classified as basic imputation techniques that do not take time into account are replaced by a constant value, which can be some descriptive measure of position (mean, median or mode) of each column in which the missing values are located [50, 51]. Now for the basic techniques that take time into account, such as time series, there are the techniques of forward fill, back fill and linear interpolation [52]. Linear interpolation is an imputation technique that assumes a linear relationship between the observed and missing values.

Advanced methods can be classified as multivariate statistical techniques or machine learning. Advanced machine learning imputation techniques use machine learning algorithms to impute missing values in a data set. One such technique is K-nearest neighbor [53], which uses the observed values of the nearest neighbors to replace the missing value. Among the statistical techniques, we highlight the application of principal component analysis (PCA), in conjunction with the NIPALS algorithm [33] and the EM algorithm [38].

### Principal component analysis

Principal component analysis (PCA), introduced by Karl Pearson [54] and based on Hotelling [55], aims to reduce the dimension of a data set by explaining the variance and covariance structure of a random vector made up of p random variables, by constructing new variables obtained by linearly combining the original variables. These linear combinations are called principal components and are not correlated with each other [56]. PCA traditionally seeks to find the directions of maximum variance in the data and represents each observation in terms of these directions, principal components. However, in incomplete data sets, conventional PCA requires association with other algorithms, such as NIPALS [33] and EM [38], i.e. NIPALS-PCA and EM-PCA, respectively.

### NIPALS-PCA

NIPALS-PCA is an extension of PCA that uses the NIPALS algorithm to find the principal components. The NIPALS algorithm is iterative and calculates the principal components one at a time using the partial least squares technique. This allows it to deal with non-linearities in the data and is especially useful in data sets with high dimensionality or complex correlations between variables. Its ability to deal with these complexities makes it a valuable tool for exploratory analysis and data modeling. This work makes use of the NIPALS-PCA algorithm implemented in the ***R-Gui*** computing environment, NIPALS package [33].

### EM-PCA

The EM-PCA iterative method [57] seeks to minimize the least squares criterion in the observed inputs. Minimization is achieved through an iterative procedure, missing values are replaced by random values. PCA is then applied to the completed data set and the missing values are updated by the fitted values using a predefined number of dimensions. This procedure is repeated until convergence [44]. This method provides estimates for individuals and variables, and an imputation for missing values. An important question concerns the number of dimensions that must be defined at the start of the iterative algorithm. The researchers Josse and Husson [58] suggested methods based on cross-validation to estimate this parameter from an incomplete data set. The method is implemented in the R-Gui computing environment, using the imputePCA function from the missMDA package [23, 39]. Further details can be found in the works [38, 41, 42, 57, 59].

### Number of components

The *missMDA* package [39] of the *R-Gui* computing environment provides functions for calculating the number of components (*estim_ncpPCA*) and some imputation methods, including EM-PCA (*imputePCA* function). For *NIPALS-PCA*, the *nipals* function from the *nipals* package was used. [33] implemented in ***R***.

Cross-validation was used to define the number of components to be used in imputation via PCA [23, 60] using the kfold method [61–63]. The percentage of missing values (*pNA*) is removed and estimated with an EM-PCA model using the range of dimensions [*ncp*.*min*, *ncp*.*max*]. This process was repeated *nbsim* times. Each cell is estimated using the *imputePCA* function, i.e. using the iterative PCA algorithm (*EM cross-validation*). The number of components resulting in the lowest mean square error was set as the number of components for imputation.

### *Missing* scenarios

Five *miss* scenarios were simulated (10%, 20%, 30%, 40% and 50%), using the mechanism *Missing Completely at Random*, MCAR [64]. To create each *missings* scenario, a data set was randomly generated with some positions taken from the ET database. This procedure begins with the random generation of seeds with the *sample* function of the basic ***R*** package, for each specific seed (*set*.*seed*) the random positions of the *missings were* generated, again with the *sample* command, according to a specific rate of missing data. For all positions, the observed values were replaced by the value *"NA"*, i.e. missing data. For each *missings* scenario specified, imputations were made using the NIPALS-PCA, EM-PCA and IM procedures. This cycle was run 100 times and, at the end, the average performance indicators were calculated.

### Statistical performance evaluation

The following indicators were used to assess the statistical performance of the NIPALS-PCA, EM-PCA and IM imputation procedures: correlation coefficient (r) [65–67], Mean Absoluto Error (MAE) [68–70], Mean Absolute Percentage Error (MAPE) [68], Mean Square Error (MSE) [71–73], Root Mean Square Error (RMSE) [67, 68, 70, 72–75], Normalized Root Mean Square Error (nRMSE) [66, 68], Willmott Index (d) [67, 73, 76] e o performance index (c) [77]. The indicators can be calculated by Eq (2) through Eq (9):

$$r=\frac{\sum_{i=1}^{m}\left(x_{i}-\bar{x}\right)\left(y_{i}-\bar{y}\right)}{\sqrt{\sum_{i=1}^{m}{\left(x_{i}-\bar{x}\right)}^{2}\sum_{i=1}^{m}{\left(y_{i}-\bar{y}\right)}^{2}}}$$ (2)

$$MAE=\frac{1}{m}\sum_{i=1}^{m}\left|x_{i}-y_{i}\right|$$ (3)

$$MAPE=100*\frac{1}{m}\sum_{i=1}^{m}\left|\frac{x_{i}-y_{i}}{x_{i}}\right|$$ (4)

$$MSE=\frac{1}{m}\sum_{i=1}^{m}{\left(x_{i}-y_{i}\right)}^{2}$$ (5)

$$RMSE=\sqrt{\frac{1}{m}\sum_{i=1}^{m}{\left(x_{i}-y_{i}\right)}^{2}}$$ (6)

$$nRMSE=100*\frac{RMSE}{\frac{1}{m}\sum_{i=1}^{m}x_{i}}$$ (7)

$$d=1-\frac{\sum_{i=1}^{m}{\left(x_{i}-y_{i}\right)}^{2}}{\sum_{i=1}^{m}{\left(\left|x_{i}-\bar{x}\right|+\left|y_{i}-\bar{x}\right|\right)}^{2}}$$ (8)

c=r*d (9)

Where “*x*_*i*_” is i-*th* observed value (*i = 1*, *…*., *m*), “$\bar{x}$” is average of observed values, “*y*_*i*_” is i-*th* imputed value (*i = 1*, *…*., *m*), “$\bar{y}$” *is* average of imputed values, “*m” is* number of missings.

## Results and discussion

In the missing data scenarios (10%, 20%, 30%, 40%, 50%) simulated in the ET database, Principal Component Analysis was used using the NIPALS (with 45 components) and EM (with 5 components) algorithms and simple mean imputation (MI) to reconstruct the database and, consequently, obtain the estimated values of the simulated missings. Seven performance measures (*r*, *MAE*, *MAPE*, *MSE*, *nRMSE*, *d* and *c*) were implemented to evaluate the performance of the methods: NIPALS-PCA, EM-PCA and IM.

### Dispersion and the correlation coefficient

**Fig 3:**
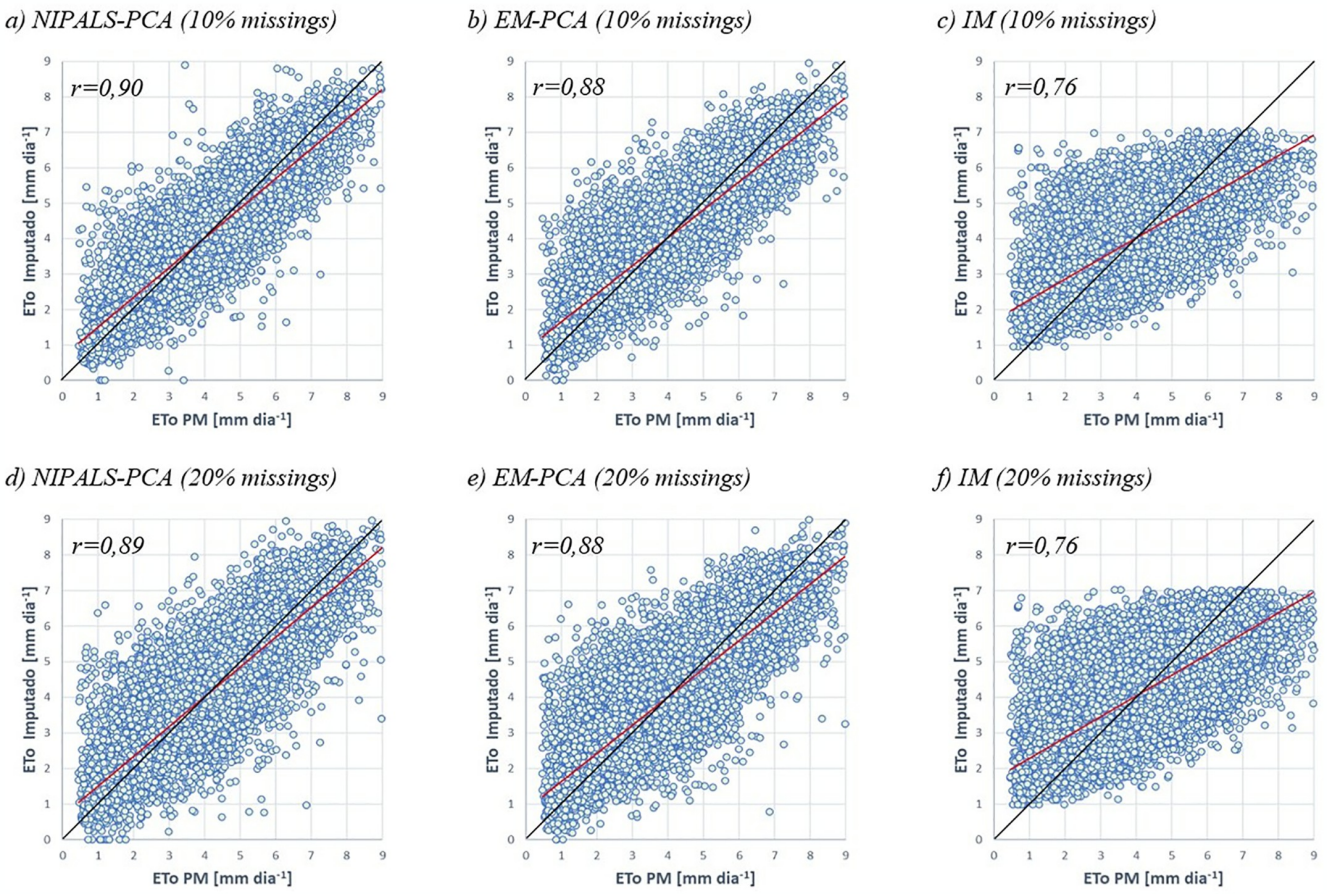
Dispersion between observed ET_o_ values and those imputed by the NIPALS-PCA, EM-PCA and IM methods (10% and 20% scenarios). **Source**: Own authorship.

**Fig 4:**
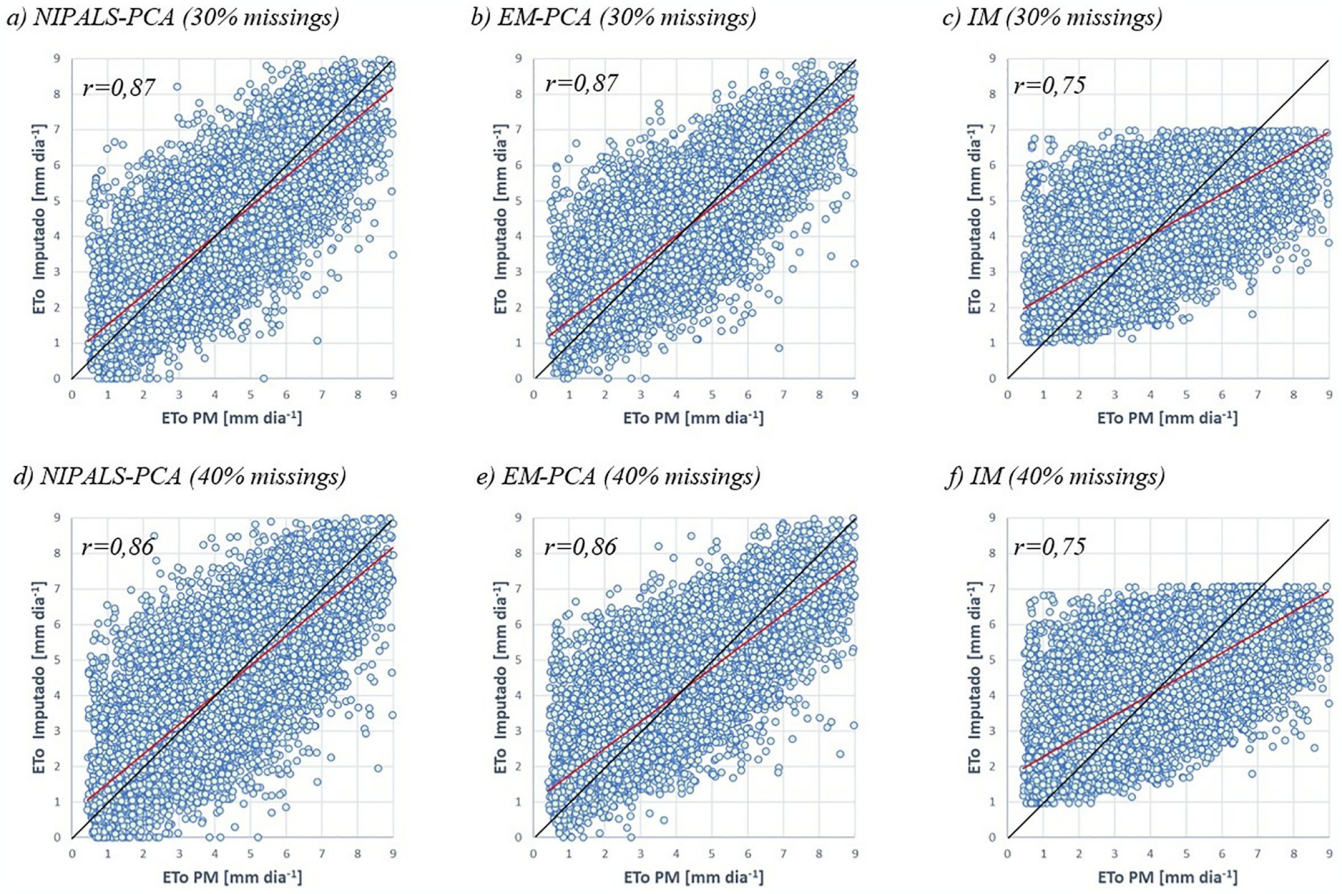
Dispersion between observed ET_o_ values and those imputed by the NIPALS-PCA, EM-PCA and IM methods (30% and 40% scenarios). **Source**: Own authorship.

**Fig 5:**
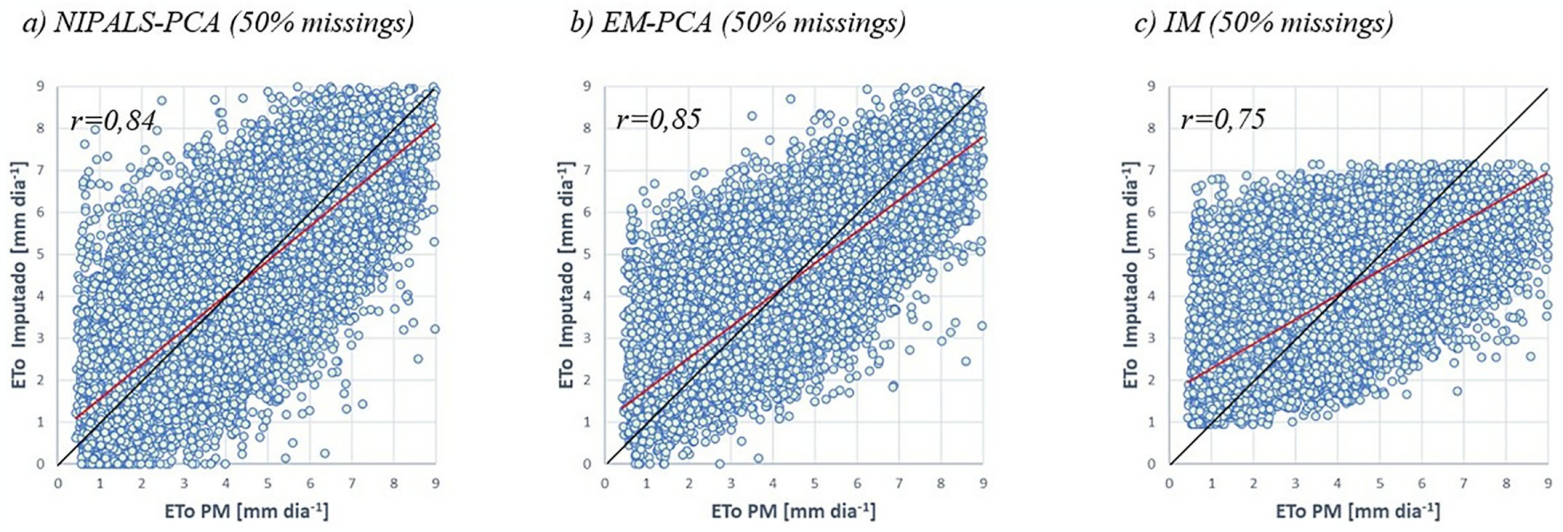
Dispersion between observed ET_o_ values and those imputed by the NIPALS-PCA, EM-PCA and IM methods (50% scenario). **Source**: Own authorship.

Figs 3–5 show the dispersion of the ordered pairs corresponding to the imputed values (by NIPALS-PCA, EM-PCA and IM) and the observed ETo values in a typical simulation. When a point coincides with the ideal line, represented by the black curve, this indicates that the estimate and the observed value are corresponding. If the point is above this curve, it means that the imputed value is underestimated; when it is below, it means that it is overestimated. The curve in red represents a linear regression of the cloud of estimated versus observed points, and it is important to note that the deviation from this curve indicates the performance of the model in question. Like the points, when the red line is above the black line, it indicates underestimated estimates, and when it is below, it indicates overestimated estimates. As can be seen in Figs 3–5, the IM imputation method visually shows a greater distance between the ideal and observed lines compared to the NIPALS-PCA and EM-PCA methods.

In Fig 3, two scenarios of missing data are presented, with rates of 10% and 20%. It is observed that the correlation coefficients for the EM-PCA and IM methods remain unchanged, at 0.88 and 0.76, respectively. Conversely, the NIPALS method shows superior results, with correlation coefficients of 0.90 and 0.89 for the 10% and 20% cases, respectively.

In Fig 4, the NIPALS-PCA and EM-PCA methods show similar results, with correlation coefficients of 0.87 and 0.86 for the 30% and 40% scenarios, respectively, while the IM method shows a result of 0.75.

Fig 5 shows the scenario of 50% missing data, estimated by the NIPALS-PCA, EM-PCA and IM methods. In this case, there was an inversion between the methods in terms of correlation coefficient, with values of 0.85 and 0.84 for EM-PCA and NIPALS-PCA, respectively.

### Analysis of performance indicators

A descriptive summary of the results obtained by performance indicators in simulations for each scenario and imputation procedure can be observed in Tables 2–5, which include the mean, minimum, and maximum values.

**Table 2:** Correlation coefficient (10%, 20%, 30%, 40% and 50% scenarios).

Indicators	% *missings*	NIPALS-PCA	EM-PCA	IM
*r*	***10***	**0,90** [0,89; 0,91]	**0,88** [0,88; 0,89]	**0,76** [0,75; 0,76]
	***20***	**0,89** [0,88; 0,89]	**0,88** [0,88; 0,89]	**0,76** [0,75; 0,76]
	***30***	**0,87** [0,87; 0,88]	**0,87** [0,86; 0,88]	**0,75** [0,75; 0,76]
	***40***	**0,86** [0,85; 0,86]	**0,86** [0,86; 0,86]	**0,75** [0,75; 0,76]
	***50***	**0,84** [0,83; 0,84]	**0,85** [0,85; 0,86]	**0,75** [0,75; 0,75]

**Source**: Own authorship.

*Average; [Minimum value, Maximum value]

**Table 3:** MAE and MSE indicators (10%, 20%, 30%, 40% and 50% scenarios).

Indicators	% *missings*	NIPALS-PCA	EM-PCA	IM
*MAE* [mm day^-1^]	***10***	**0,50** [0,49; 0,51]	**0,53** [0,52; 0,54]	**0,75** [0,73; 0,76]
	***20***	**0,53** [0,53; 0,54]	**0,54** [0,54; 0,55]	**0, 75** [0,74; 0,76]
	***30***	**0,57** [0,56; 0,57]	**0,58** [0,55; 0,59]	**0, 75** [0,75; 0,76]
	***40***	**0,61** [0,60; 0,61]	**0,60** [0,59; 0,60]	**0, 76** [0,75; 0,76]
	***50***	**0,65** [0,65; 0,66]	**0,60** [0,60; 0,61]	**0, 76** [0,76; 0,77]
*MSE* [mm^2^ day^-2^]	***10***	**0,48** [0,45; 0,51]	**0,54** [0,52; 0,56]	**1,07** [1,03; 1,11]
	***20***	**0,55** [0,53; 0,58]	**0,56** [0,54; 0,58]	**1,07** [1,05; 1,10]
	***30***	**0,62** [0,60; 0,64]	**0,62** [0,57; 0,66]	**1,08** [1,06; 1,10]
	***40***	**0,70** [0,68; 0,72]	**0,66** [0,65; 0,67]	**1,08** [1,06; 1,11]
	***50***	**0,80** [0,78; 0,83]	**0,69** [0,67; 0,69]	**1,09** [1,08; 1,11]

**Source**: Own authorship.

*Average; [Minimum value, Maximum value]

**Table 4:** MAPE and nRMSE indicators (10%, 20%, 30%, 40% and 50% scenarios).

Indicators	% *missings*	NIPALS-PCA	EM-PCA	IM
*MAPE* [%]	***10***	**15,44** [14,95; 15,89]	**16,96** [16,53; 17,43]	**24,65** [23,90; 25,43]
	***20***	**16,40** [16,04; 16,72]	**17,15** [16,83; 17,54]	**24,74** [24,26; 25,23]
	***30***	**17,46** [17,16; 17,72]	**18,29** [17,18; 18,98]	**24,84** [24,42; 25,23]
	***40***	**18,59** [18,29; 18,85]	**18,89** [18,63; 19,15]	**24,93** [24,60; 25,26]
	***50***	**19,92** [19,65; 20,37]	**19,13** [18,91; 19,29]	**25,06** [24,76; 25,28]
*nRMSE* [%]	***10***	**17,16** [16,60; 17,60]	**18,18** [17,78; 18,55]	**25,50** [25,03; 26,01]
	***20***	**18,27** [17,96; 18,70]	**18,41** [18,15; 18,76]	**25,55** [25,24; 25,89]
	***30***	**19,41** [19,18; 19,65]	**19,46** [18,58; 20,01]	**25,61** [25,41; 25,89]
	***40***	**20,62** [20,31; 21,01]	**20,03** [19,85; 20,19]	**25,67** [25,45; 25,94]
	***50***	**22,07** [21,77; 22,41]	**20,30** [20,14; 20,45]	**25,77** [25,58; 25,96]

**Source**: Own authorship.

*Average; [Minimum value, Maximum value]

**Table 5:** Willmott and performance indicators (10%, 20%, 30%, 40% and 50% scenarios).

Indicators	% *missings*	NIPALS-PCA	EM-PCA	IM
**d**	***10***	**0,95 [0,94; 0,95]**	**0,94 [0,93; 0,94]**	**0,85 [0,84; 0,86]**
	***20***	**0,94** [0,94; 0,94]	**0,94** [0,93; 0,94]	0,**85** [0,85; 0,86]
	***30***	**0,93** [0,93; 0,93]	**0,93** [0,92; 0,93]	0,**85** [0,85; 0,85]
	***40***	**0,92** [0,92; 0,93]	**0,92** [0,92; 0,92]	0,**85** [0,85; 0,85]
	***50***	**0,91** [0,91; 0,92]	**0,92** [0,92; 0,92]	**0,85** [0,85; 0,85]
**c**	***10***	**0,85** [0,84; 0,86]	**0,83** [0,82; 0,84]	**0,65** [0,64; 0,66]
	***20***	**0,83** [0,82; 0,84]	**0,82** [0,82; 0,83]	**0,64** [0,64; 0,65]
	***30***	**0,81** [0,81; 0,82]	**0,80** [0,79; 0,82]	**0,64** [0,64; 0,65]
	***40***	**0,79** [0,78; 0,80]	**0,79** [0,79; 0,79]	**0,64** [0,64; 0,65]
	***50***	**0,77** [0,76; 0,77]	**0,79** [0,78; 0,79]	**0,64** [0,64; 0,64]

**Source**: Own authorship.

*Average; [Minimum value, Maximum value]

Regarding the correlation coefficient, in Table 2, no significant differences were observed between the missing data scenarios for the IM method. For all missing data scenarios, the correlation coefficient ranged from 0.75 to 0.76 for the IM method, except for 50% missing data, where it was observed that the value practically did not vary between the simulations conducted.

The NIPALS-PCA method has an amplitude of 0.06, which is greater than that of the EM-PCA (0.03). However, in terms of average correlation coefficient, there is practically no difference between the two methods.

The Table 3 presents the results for the MAE and MSE indicators. Once again, it is observed that for the IM method, the results of the MAE and MSE indicators practically do not undergo changes. As for the NIPALS-PCA and EM-PCA methods, there is a reversal in performance: the NIPALS-PCA shows better performance in the scenarios of 10% and 20%, while the EM-PCA performs better in the scenarios of 40% and 50%.

In Table 4, the results for the MAPE and nRMSE indicators are presented. It is observed that the NIPALS-PCA method outperforms the EM-PCA for the scenarios of 10%, 20%, and 30%. However, there is a performance reversal in the 50% scenario. A gradual increase in MAPE and nRMSE was observed as a greater number of missing values were added, indicating a deterioration in the performance of the procedures considered. For the scenario with 10% of missing values, NIPALS-PCA obtained the lowest MAPE (15.44%), followed by EM-PCA (16.96%), while IM obtained a MAPE equal to 24.65%. In the scenario with 50% of missing values, there is a performance reversal, with a lower MAPE (19.13%) for EM-PCA, followed by NIPALS-PCA (19.92%).

Considering the classification scale for the different nRMSE intervals, the NIPALS-PCA and EM-PCA approaches present good results (10% ≤ nRMSE < 20%) in the imputation of missing values. Particularly noteworthy is the NIPALS-PCA method for the scenarios of 10%, 20%, and 30%, and the EM-PCA for the scenarios of 40% and 50%.

In Table 5, the results for the performance indices (c) and Willmott’s agreement index (d) are presented. It is observed that, for Willmott’s agreement index (d), the NIPALS-PCA and EM-PCA methods stand out, on average, with an agreement index of 93%, compared to 85% for the IM method. Regarding the confidence coefficient (c), the NIPALS-PCA and EM-PCA methods present an average value of 0.81, classified as "very good" estimation models according to the classification provided by researchers Camargo and Sentelhas [77]. On the other hand, the IM method showed an average value of 0.64, classified as a "good" estimation model.

**Fig 6:**
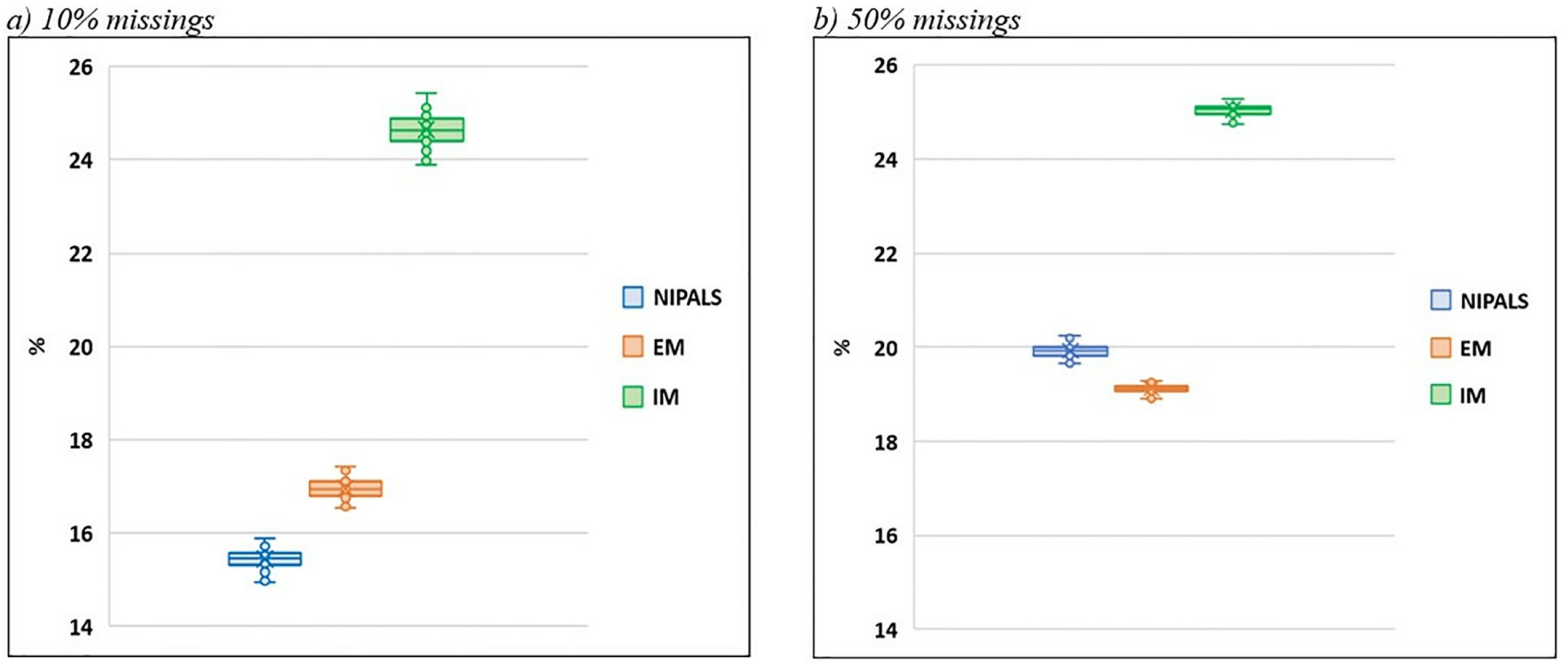
MAPE indicator (10% and 50% scenarios). **Source**: Own authorship.

Fig 6 presents the results for the MAPE indicator for the scenarios of 10% and 50%. It can be observed that the EM-PCA and NIPALS-PCA methods are similar, while the IM method deviates, showing results with higher deviations.

### Research comparison

Compared to the results obtained by researchers Martí and Zarzo [24] modeling 30 weather stations located in the Valencia region of Spain, from 2000 to 2007, we see lower results in the performance indicators than those found in this research, as shown in Table 6, for the 10% *missings* scenario.

**Table 6:** Values of some statistical performance indicators.

Researchers	Location	MSE mm^2^ day^-2^	MAE mm day^-1^	pMAE %	r
Martí and Zarzo (2012)	Valencia—Spain	0,11	0,24	9,20	0,98
Present research	São Paulo—Brazil	0,48	0,50	15,44	0,90

**Source**: Own authorship.

Researchers Dray and Josse [44] review some PCA imputation methods applied to data in the field of ecology. They suggest using EM-PCA rather than NIPALS-PCA, due to the difficulty of convergence. This was not observed in this study.

### Research limitations

It is important to note that the simulations carried out in this study used the *Missing Completely at Random* (MCAR) mechanism and, therefore, the results presented may not be generalizable to situations in which the missing values occurred in a non-random or biased manner. In addition, this study used the mean to complete the initial base, favoring this method in the simulations carried out and, therefore, the differences between the performance of the multivariate methods via PCA in relation to imputation by the mean may be greater.

## Conclusions

This study examined the performance of alternative multivariate principal component analysis procedures using NIPALS and EM algorithms, along with simple mean imputation (IM), for reconstructing a high-dimensional, small-sample reference evapotranspiration database. Consequently, estimated values for simulated missing data were obtained under scenarios of 10%, 20%, 30%, 40%, and 50% missing data. The study spanned from 2012 to 2021 and focused on automatic weather stations in the São Paulo region, Brazil. Results underscored the importance of choosing the right imputation approach, with significant implications for the accuracy of climate estimates. PCA proved to be a useful tool for estimating missing values, particularly when the sample size was small relative to the number of variables. This study focused on imputing missing data in an evapotranspiration database, considering ET_o_ measurement days as correlated variables (3653 columns) measured across 45 automatic weather stations (rows). Statistical performance comparison among the techniques revealed that NIPALS-PCA and EM-PCA outperformed IM, depending on the percentage of missing data. Based on the statistical indicator classification of the validation base for NIPALS-PCA, EM-PCA, and IM imputation models, there are indications that they are suitable for estimating missing reference evapotranspiration values, with particular emphasis on PCA imputation models in the NIPALS and EM algorithms. For future work, exploring the effectiveness of different imputation methods across various climatic and geographic contexts is recommended. Investigations into the development of new imputation techniques, especially those considering the temporal and spatial structure of meteorological data, are essential for advancing understanding and forecasting capacity in climatology. In summary, this study provides a solid foundation for future research on imputation strategies for missing meteorological data, with the potential to significantly improve the accuracy and utility of climate estimates in various applications.
